# The role of parents on hand hygiene promotion in the pediatric setting – a systematic review

**DOI:** 10.1186/2047-2994-4-S1-P290

**Published:** 2015-06-16

**Authors:** F Bellissimo-Rodrigues, W Zingg, D Pittet

**Affiliations:** 1 Infection Control Programme, University of Geneva Hospitals, Switzerland; 2 Collaborating Centre on Patient Safety, World Health Organization (WHO), Geneva, Switzerland

## Introduction

The parental instinct of offspring protection has evolved and perpetuated for millenniums across a wide range of species. When a child is sick and particularly when it is hospitalized, parents and other caregivers have to share their natural and social role of child protection with the healthcare workers. This may create conflict but also the opportunity for establishing a partnership between all actors in favor of delivering safe care to the child. One of the main issues on that partnership is the promotion of hand hygiene (HH) because both parents and healthcare workers must respect HH, which is considered the most effective preventive measure against health-care associated infections (HAI).

## Objectives

We aimed to review the scientific evidence available about the parents’ participation in hand hygiene promotion in the pediatric setting.

## Methods

We performed a systematic search in Pubmed using the following search term: [(“hand hygiene” OR “hand wash*” OR “hand rub*”) AND (“caregiver*” OR “parent*” OR “mother*” OR “father*” OR “family”)], All quantitative and qualitative studies addressing the role of caregivers in HH promotion and published until 31 December 2014 were eligible.

## Results

We found 86 articles in the system, but only 5 original articles addressed the study question. All studies were observational and used questionnaires or interviews. Their main findings were as follows: most parents are aware about the risk of HAI; parents recognize HH as a relevant tool for HAI prevention; their knowledge on the HH indications (“5 moments”) and on the efficacy of alcohol-based handrub was variable, however, as well as their willingness to remind healthcare workers about a HH opportunity when HH was not performed. One study found that parents felt more prone to actively intervene on HH promotion if they received a formal invitation to do so from healthcare workers.

## Conclusion

Literature on the subject is very scarce and we found no randomized clinical trial addressing it. Parents’ promotion of HH should be further explored by research, as a potential intervention for enhancing patient safety on the pediatric setting.

## Disclosure of interest

None declared.

